# Editorial: EBV Infection and Human Primary Immune Deficiencies

**DOI:** 10.3389/fimmu.2020.00130

**Published:** 2020-02-07

**Authors:** Jeffrey I. Cohen, Isabelle Meyts

**Affiliations:** ^1^Laboratory of Infectious Diseases, National Institute of Allergy and Infectious Diseases, National Institutes of Health, Bethesda, MD, United States; ^2^Department of Microbiology and Immunology, University Hospitals Leuven, Leuven, Belgium

**Keywords:** Epstein-Barr virus, immune deficiency, lymphoproliferative disease, hemophagocytic lymphohistiocytosis, B-cell lymphoma

Epstein-Barr virus (EBV) is a human herpes virus that infects nearly 95% of individuals worldwide and most persons are unaware that they are infected and never have disease associated with the infection. If infection is delayed until adolescence or early adulthood, most of these persons will develop infectious mononucleosis. Persons with certain congenital immunodeficiencies [e.g., X-linked lymphoproliferative disease 1 (XLP1)], acquired immunodeficiencies (e.g., AIDS), or iatrogenic immunodeficiencies (e.g., organ transplant recipients) can develop severe or even fatal EBV disease associated with primary infection or reactivation of the virus. These diseases include EBV B cell or T cell lymphoma, lymphoproliferative disease (LPD), hemophagocytic lymphohistiocytosis (HLH), or EBV smooth muscle tumors. Genetic disorders associated with severe EBV disease include those that predispose to infections with multiple viruses, bacteria, or fungi (e.g., GATA2 deficiency) or infection primarily associated with EBV alone (XLP1). These disorders primarily affect the function of T cells and NK cells which are important for immune surveillance against EBV-infected cells, rather than B cells that the virus infects, establishes latency in, and can drive to LPD. Identification of genetic disorders associated with EBV has furthered our knowledge of the role of the functions of cellular proteins important for signaling and effector activity of T cells and NK cells.

The collection of articles on EBV Infectious and Human Primary Immune Deficiencies begins with an overview of T cell responses to the virus by Long et al. This review emphasizes the importance of T cell responses to EBV during symptomatic and asymptomatic primary infection and during persistent infection. The authors also describe the contributions of tissue resident memory T cells, γδ T cells, and NKT cells for control of EBV infection. Latour and Winter provide an overview of immune deficiencies that predispose to EBV LPD. These include mutations in proteins that impair T cell proliferation, B cell-T cell interactions, and T cell and NK-cell cytotoxicity. Additional articles in this collection focus on specific immune deficiencies associated with severe EBV disease. Ghosh et al. report on IL-2 inducible kinase (ITK) deficiency which is critical for T cell signaling. Patients with defects in ITK can present with EBV-positive Hodgkin and non-Hodgkin lymphoma, LPD, and HLH. Panchal et al. review findings in patients with XLP1 who have loss-of-function mutations in SAP that present with B cell lymphoma, HLH, and/or dysgammaglobulinemia. SAP is an adapter protein important for activation of SLAM family members and signaling in T and NK cells. Patients with mutations in SAP have impaired T and NK cell function. Arjunaraja et al. report on B cell expansion with NF-κB and T cell anergy (BENTA) disease which is associated with gain-of-function mutations in CARD11. These patients have B cell lymphocytosis, reduced numbers of T and NK cells, low grade persistent EBV viremia, and constitutive activation of NF-κB. Caorsi et al. describe a patient with CD70 deficiency who presented with periodic fever, tonsillitis, cervical lymphadenitis, and EBV viremia. CD70 is expressed on antigen presenting cells (including B cells) and is the ligand for CD27 which is expressed on T cells; this interaction is important for cytotoxic T cell activation. Hoeger et al. report that nuclear factor kappa-light-chain-enhancer of activated B cells 1 (NF-κB1) haploinsufficiency is associated with common variable immunodeficiency-like B cell disease, recurrent pulmonary infections, and EBV LPD. NF-κB1 is important for NF-κB signaling in both cytotoxic T cells and in B cells. Carpier and Lucas review activated PI3Kδ syndrome (APDS) which is due to gain-of-function mutations in *PI3K3CD* or *PIK3R* or loss-of-function mutations in *PTEN*. These mutations result in constitutive activation of PI3K with senescent CD8 T cells and increased numbers of terminal effector CD8 T cells. Patients present with frequent sinopulmonary infections, EBV viremia, LPD, and lymphoma, as well as cytomegalovirus viremia and lymphadenitis. Kimura and Cohen describe chronic active EBV disease in which patients have high levels of EBV in circulating T or NK cells (or less commonly in B cells) which infiltrate the tissues and often result in EBV lymphoma or HLH. Some of these patients have somatic mutations in their EBV-positive T or NK cells, usually associated with driver mutations in genes such as *DDX3X* and *BCOR*.

Patients with genetic disorders associated with severe EBV can develop HLH. Marsh reports that patients with HLH present with fever, splenomegaly, reduced numbers of erythrocytes, leukocytes, or platelets, and often hepatitis. Cytotoxic T cells or NK cells from patients with HLH have impaired degranulation or cytotoxicity, and persistent hyperinflammation is present. HLH with severe EBV disease has been associated with mutations in *SH2D1A, BIRC4, CD27, ITK*, and *MAGT1*. While EBV-positive smooth muscle tumors were initially reported in solid organ transplant recipients or patients with AIDS, Magg et al. report that these tumors have been reported in immune deficiencies associated with EBV, including GATA2 or CARMIL2 deficiency, ataxia telangiectasia, and severe combined immune deficiency associated with mutations in *ADA, ZAP70*, or *IL2RG*. While hematopoietic stem cell transplantation (HSCT) has been used to correct many EBV-associated genetic disorders, many of these patients have severe viral infections prior to transplant which increases the morbidity associated with HSCT, and some may have relapses of EBV disease after HSCT. McLaughlin et al. report that the use of EBV-specific cytotoxic T cells either before HSCT to gain better control of infections, or after transplant to treat persistent EBV disease, has been effective. EBV-specific T cells derived from the HSCT donor or third-party HLA-matched cells have been effective.

The articles in this collection describe many of the genetic disorders associated with EBV; new disorders continue to be discovered. These diseases continue to inform us about the importance of interactions between T or NK cells and EBV-infected B cells and how the only human virus that establishes latency in B cells and induces B cell lymphoproliferation is controlled by our immune system. Better understanding of the role of individual T and NK cell proteins in controlling EBV may lead to improved immunologic-based treatments for both EBV disease as well as for cancer. In addition, identification of key proteins important for T cell and NK cell function could lead to novel targets for immune suppressive medications.

## Author Contributions

All authors listed have made a substantial, direct and intellectual contribution to the work, and approved it for publication.

### Conflict of Interest

The authors declare that the research was conducted in the absence of any commercial or financial relationships that could be construed as a potential conflict of interest.

